# Role of NADPH Oxidase-4 in Human Endothelial Progenitor Cells

**DOI:** 10.3389/fphys.2017.00150

**Published:** 2017-03-23

**Authors:** Nora Y. Hakami, Amaresh K. Ranjan, Anandwardhan A. Hardikar, Greg J. Dusting, Hitesh M. Peshavariya

**Affiliations:** ^1^Centre for Eye Research Australia, Royal Victorian Eye and Ear HospitalEast Melbourne, VIC, Australia; ^2^Ophthalmology, University of Melbourne, Department of SurgeryEast Melbourne, VIC, Australia; ^3^Department of Pharmacology and Therapeutics, University of MelbourneMelbourne, VIC, Australia; ^4^Faculty of Applied Medical Sciences, King Abdulaziz UniversityJeddah, Saudi Arabia; ^5^Cardiology, Icahn School of Medicine at Mount Sinai HospitalNew York, NY, USA; ^6^Diabetes and Islet Biology, NHMRC Clinical Trials Centre, University of SydneySydney, NSW, Australia

**Keywords:** NOX4, H_2_O_2_, endothelial progenitor cells, ROS, TNFα

## Abstract

**Introduction:** Endothelial progenitor cells (EPCs) display a unique ability to promote angiogenesis and restore endothelial function in injured blood vessels. NADPH oxidase 4 (NOX4)-derived hydrogen peroxide (H_2_O_2_) serves as a signaling molecule and promotes endothelial cell proliferation and migration as well as protecting against cell death. However, the role of NOX4 in EPC function is not completely understood.

**Methods:** EPCs were isolated from human saphenous vein and mammary artery discarded during bypass surgery. NOX4 gene and protein expression in EPCs were measured by real time-PCR and Western blot analysis respectively. NOX4 gene expression was inhibited using an adenoviral vector expressing human NOX4 shRNA (Ad-NOX4i). H_2_O_2_ production was measured by Amplex red assay. EPC migration was evaluated using a transwell migration assay. EPC proliferation and viability were measured using trypan blue counts.

**Results:** Inhibition of NOX4 using Ad-NOX4i reduced Nox4 gene and protein expression as well as H_2_O_2_ formation in EPCs. Inhibition of NOX4-derived H_2_O_2_ decreased both proliferation and migration of EPCs. Interestingly, pro-inflammatory cytokine tumor necrosis factor alpha (TNFα) decreased NOX4 expression and reduced survival of EPCs. However, the survival of EPCs was further diminished by TNF-α in NOX4-knockdown cells, suggesting that NOX4 has a protective role in EPCs.

**Conclusion:** These findings suggest that NOX4-type NADPH oxidase is important for proliferation and migration functions of EPCs and protects against pro-inflammatory cytokine induced EPC death. These properties of NOX4 may facilitate the efficient function of EPCs which is vital for successful neovascularization.

## Introduction

Neovascularization is critical for normal development and in wound repair (Bauer et al., 2005; Fish and Wythe, 2015). It is also seen as a potential option to rescue tissue from critical damage after acute ischemia such as myocardial infarction (Isner and Asahara, 1999). Endothelial progenitor cells (EPCs) are a type of cell able to differentiate into mature endothelial cells and are involved in postnatal neovascularization (Takahashi et al., 1999; Asahara et al., 1999a,b; Aicher et al., 2005; Real et al., 2008). Evidence continues to accumulate regarding the importance of EPCs for restoring endothelial function in injured blood vessels and neovascularization in ischemic tissues (Asahara et al., 1999a; Assmus et al., 2002; Iba et al., 2002; Griese et al., 2003; Krankel et al., 2012; Galasso et al., 2013; Lee et al., 2016).

Although currently there is no defining characteristic of EPCs, these cells are primarily identified by expression of haematopoietic stem cell surface antigens CD133, CD34, and Vascular endothelial growth factor receptor 2 (VEGFR2) (Yin et al., 1997; Peichev et al., 2000; Urbich and Dimmeler, 2004; Friedrich et al., 2006; Zengin et al., 2006; Chang et al., 2007; Chen et al., 2007; Ranjan et al., 2009). Additionally, EPCs take up acetylated low-density lipoprotein conjugated 1,1′-dioctadecyl- 3,3,3′,3′-tetramethylindocarbocyanine (Di-Ac-LDL) and bind to ulex europaeus agglutinin-1 (UEA-1) lectin. EPCs are classified as either early endothelial colony forming cells (ECFC)or late outgrowth endothelial cells (OEC or EOC) based on *in vitro* growth and survival (Asahara et al., 2011). Peak growth is observed in early haematopoietic EPCs, characteristically spindle shaped, at 2–3 weeks with death occurring by 4 weeks. Late EPCs, on the other hand, are cobblestone-like in morphology within 2–3 weeks, demonstrate exponential growth between 4 and 8 weeks and can survive up to 12 weeks (Marui et al., 1993; Asahara et al., 1997; Hur et al., 2004). Furthermore, early EPCs do not proliferate *in vitro* (Rehman et al., 2003) whereas, late EPCs are highly proliferative, differentiate into mature endothelial cells and are directly incorporated into blood vessels during neovascularization (Hur et al., 2004; Ranjan et al., 2009; Cheng et al., 2013).

Reactive oxygen species (ROS) including superoxide and hydrogen peroxide (H_2_O_2_) act as double-edged swords in pathophysiological conditions. ROS can be either protective or destructive, depending upon the particular species (e.g., superoxide vs. H_2_O_2_), their location, and the amounts generated. NADPH oxidases (NOX) are the major source of ROS and are involved in modulation of stem or progenitor cell function under various conditions (Imanishi et al., 2008; Schröder et al., 2009; Ushio-Fukai and Urao, 2009; Turgeon et al., 2012; Peng et al., 2015). The NOX family consists of 7 isoforms NOX1-5 (NOX5 is only expressed in humans) and dual oxidases (DUOX) 1 and 2 (Bedard and Krause, 2007; Brandes and Ushio-Fukai, 2011). Endothelial cells and EPCs predominantly express NOX2, NOX4, and NOX1 isoforms of NADPH oxidase (Piccoli et al., 2007). Each isoform differs in terms of expression, subcellular localization, type of ROS produced and their activation (Chan et al., 2009; Drummond et al., 2011). For instance, NOX2 isoform requires growth factors (vascular endothelial growth factor; VEGF, and hepatocyte growth factor; HGF) or cytokines (tumor necrosis factor alpha; TNFα and angiotensin-II; Ang II), recruiting cytosolic subunits for full activation to produce superoxide (Jiang et al., 2004; Cave et al., 2006; Bedard and Krause, 2007; Frey et al., 2009; Brandes and Ushio-Fukai, 2011). There is increasing evidence to suggest that NOX2-mediated ROS signaling can modulate EPC function. For instance it has been shown that NOX2-derived ROS signaling promotes the mobilization and angiogenic capacities of bone marrow derived-early EPCs, that can contribute to revascularization of ischemic tissue (Urao et al., 2008) and re-endothelialization of injured arteries (Urao et al., 2008; Schröder et al., 2009, 2011). On the other hand, sustained overproduction of NOX2-derived superoxide (i.e., oxidative stress) causes EPC dysfunction and impairment of neovascularization under various pathological conditions including heart failure, diabetes and hypertension (Ebrahimian et al., 2006; Yao et al., 2007; Hamed et al., 2009). Mechanistically, NOX2 derived superoxide reduces nitric oxide (NO) bioavailability and inhibits telomerase activity, leading to EPC senescence and dysfunction (Sorrentino et al., 2007; Hamed et al., 2011).

In contrast to other NOX isoforms, NOX4 does not require activation as it is constitutively active and primarily produces H_2_O_2_ (i.e., reductive stress) instead of superoxide in endothelial cells (Brandes et al., 2011; Takac et al., 2011; Schroder et al., 2012; Peshavariya et al., 2014b). Recently, we and others have shown that increased expression of NOX4 in endothelial cells protects the cells from death and increases neovascularization *in vitro* and *in vivo* (Schroder et al., 2012; Yan et al., 2014; Peshavariya et al., 2014b). However, the role of NOX4 in EPC function is poorly understood and the current study was undertaken to examine whether or not NOX4 plays similar role in human EPCs.

## Materials and methods

### Isolation and expansion of cells

Human endothelial progenitor cells were isolated from human mammary arteries/saphenous vein as described earlier (Ranjan et al., 2009). Briefly, 2–4 cm long pieces of human mammary artery/saphenous vein were collected as de-identified surgical waste following written informed consent from 50 to 70 years old individuals undergoing cardiac surgery. Mammary artery/saphenous vein samples were collected in M199 medium containing antibiotics (penicillin 25 unit/ml, streptomycin 25 μg/ml, Ciprofloxacin 30 μg/ml, GIBCO-BRL, Burlington, ON), then washed with PBS. Collection of such material was approved by the NCCS research ethics committee (Ethics No. NCCS06/198) National Center for Cell Science, Pune, India. EPCs were detached from the luminal wall of arteries using collagenase (1 mg/ml, Sigma, St. Louis, MO) for 15 min at 37°C. Sterile PBS were used to flush out the detached EPCs from the lumen, and collected in 15 ml Falcon tubes. EPCs were washed twice with 10 ml of PBS and then cell seeded in 10% human AB sera and antibiotics (5 unit/ml penicillin, 5 μg/ml streptomycin, 6 μg/ml Ciprofloxacin, GIBCO-BRL, Burlington, ON) containing endothelial growth medium mix or EGMM, which was made by mixing 50% M199 medium (GIBCO-BRL, Burlington, ON) with EBM-2 medium (Lonza, Walkersville, MD). Isolated EPCs adhere to culture plates within 6–8 h, after which, all non-adherent cells were removed. Adherent EPCs were expanded following procedures described earlier (Ranjan et al., 2009) and used for the present study.

### Flow cytometry staining

EPCs were harvested, washed in Ca^++^ and Mg^++^ free PBS before blocking for non-specific antigens using 4% normal donkey serum for 20 min. CD133 one step staining was carried out using phycoerythrin(PE)-tagged mouse anti-human CD133 antibody (1:50; Miltenyi Biotec, Germany) and incubation at 4°C for 45 min. Later, EPCs were washed with 5 ml PBS for three times. Cells were then re-suspended in 300 ml of PBS. Each sample was assessed for at least 10,000 events on a FACS Canto II system (BD, Franklin Lakes, NJ) and then analyzed using the FACS Diva software as described earlier (Ranjan et al., 2009).

### Cell culture

EPCs were cultured on 1% fibronectin coated dishes in EGM-2 Bullet Kit with 15% fetal bovine serum (FBS) known as EGM-2 growth medium (Lonza, VIC, Australia). EPCs were cultured under standard cell culture conditions using a 5% CO2 incubator at 37°C. Tumor necrosis factor (TNF-α) is well known to induce Nox isoforms and cell death. In some conditions, cells were treated with human TNF-α (20 ng/ml; Sigma, St. Louis, MO) for 6 or 48 h prior to cell harvest.

### Adenovirus infection

We silenced the expression of NOX4 gene using adenoviral vectors carrying small interfering RNA targeting nucleotides 418–436 from the start codon of human NOX4 (Ad-NOX4i). Briefly, 5 × 10^4^ EPCs were cultured in a 10 cm^2^ plate 1 day before infecting them with Quiet-U6 vector (Welgen) expressing the sequence against human NOX4 (nucleotides 418–436 from the start codon). The construct was ligated into a linearized adenoviral genome for subsequent generation of adenoviral vector (Ad-NOX4i) (Chen et al., 2008). Adenovirus containing green fluorescent protein gene (Adv-GFP) was used as a control and optimized multiplicity of infection as shown in Supplementary Figure S1. EPCs were infected with 1,000 MOI of Ad-GFP or Ad-NOX4 RNAi for 24 h in Opti-MEM medium (Life Technologies, Victoria, Australia), then allowed to recover for another 24 h using EGM-2 growth medium. All experiments were performed 48 h after infection.

### Dil-Ac-LDL uptake assay

EPCs (10^3^ cells/well) were seeded on 1% fibronectin coated 24-well plates in EGM-2 Bullet Kit with 5% FBS (EGM-2 growth media, Lonza, Victoria, Australia) and allowed to attach following overnight incubation. For LDL uptake assay, cultured medium was replaced with 10 μg/ml of acetylated low density lipoprotein (Ac-LDL), labeled with 1,1′-dioctadecyl– 3,3,3′,3′-tetramethyl-indocarbocyanine perchlorate (DiI-Ac-LDL, Biomedical Technologies), then cells were incubated for additional 4 h. After the end of the incubation period, the solution was aspirated and fresh growth medium was added before capturing the images using fluorescence microscopy (Zeiss AxioImager.2 microscope).

### Immunostaining

Cells were fixed in 4% fresh paraformaldehyde, permeabilized with chilled 50% methanol, blocked with 4% normal donkey serum and then incubated with specific antisera. Primary antibodies; mouse anti- PECAM-1 antibody (Chemicon, Temecula, CA, 1:100), mouse anti-VE-cadherin (Chemicon, Temecula, CA, 1:100), rabbit anti-eNOS mouse antibody (Chemicon, Temecula, CA, 1:100) and UEA1 (Ulex europeous agglutinin; Sigma, St. Louis, MO; 1:100), were incubated overnight at 4°C, washed with PBS and then incubated with the secondary antibodies; Alexa-Fluor 488, Alexa-Fluor 546 (Molecular Probes, Carlsbad, CA, 1:200) at 37°C for 1 h. We used 4′, 6-diamidino-2-phenylindole (DAPI) or propidium iodide to visualize nuclei. Slides were washed extensively with generous volumes of PBS and mounted in Vectashield mounting medium (Vector Laboratories, Burlingame, CA). Confocal images for CD31 and CD144 were obtained using a Zeiss LSM 510 laser scanning microscope. All other images obtained using Zeiss AxioImager-2 microscope fluorescence microscopy. Results presented are representative fields confirmed from at least three different biological samples.

### Gene expression analysis

Cells (10^5^ cells/well) were seeded on 6-well plates. Serum deprived cells were treated with tumor necrosis factor; TNFα (20 ng/ml) for 6 or 48 h. Total RNA from treated cells was extracted with the TRI reagent according to manufacturer's instructions (Ambion, Austin, TX, USA) and reverse-transcribed to cDNA using TaqMan high performance reverse transcription reagents (Applied Biosystems, Life Technologies, Victoria, Australia) at 25°C for 10 min, 37°C for 2 h followed by 85°C for 5 s in a Thermal cycler (BioRad-DNA Engine, Bio-Rad, New South Wales, Australia). Real-time PCR reactions were performed in a 7,300 real-time quantitative PCR system (Applied Biosystems, Life Technologies) using TaqMan Universal PCR master mix and pre-designed (off the shelf) gene specific probes and primer sets for NOX2 (Hs00166163_m1) and NOX4 (Hs01558199_m1) The cycle threshold (CT) values from all real-time PCR experiments were analyzed using ^ΔΔ^CT method. Data were normalized to glyceraldehyde 3-phosphate dehydrogenase (GAPDH; human 4326317E) and expressed as fold changes over that in the control treatment group.

### Amplex® red assay

Extracellular hydrogen peroxide (H_2_O_2_) levels were detected using Amplex® Red assay kit (Molecular Probes, Life Technologies, Victoria, Australia) according to manufacturer's instructions. EPCs (10^4^ cells/well) were seeded on 1% fibronectin coated 24-well plates. Serum-deprived cells were trypsinized and suspended in Krebs-HEPES buffer (HBSS, in mM: NaCl 98.0, KCl 4.7, NaHCO_3_ 25.0, MgSO_4_ 1.2, 137 KH_2_PO_4_ 1.2, CaCl_2_ 2.5, d-glucose 11.1 and Hepes-Na 20.0) containing Amplex® Red reagent (10 μM) and horseradish peroxidase (0.1 U/ml). Fluorescence was then measured with excitation and emission at 550 nm and 590 nm respectively, using a polarstar microplate reader (BMG Labtech, Germany) at 37°C. Fluorescence values were normalized to cell numbers determined by Alamar® Blue cell viability assays as according to manufacturer's instructions (Life Technologies, Victoria, Australia).

### Proliferation assay

EPCs (5 × 10^3^ cells/well) were seeded on 24-well plates for 24 h before serum starvation. Serum starved-cells were induced to proliferate by adding EGM-2 growth medium with 1% FBS. After 48 h, cells were trypsinized and numbers were analyzed by using trypan blue (Life Technologies, Victoria, Australia) and counted using a haemocytometer.

### Transwell migration assay

The transwell migration assay was performed using 24-well transwell permeable inserts containing polycarbonate membranes (6.5 mm diameter, 8 μm pore size, and 0.3 cm^3^ bottom area, CLS3422; Sigma-Aldrich). Inserts were incubated with 0.1% gelatin and dried. Then, 600 μl of the chemo attractant (EGM-2 growth medium with 1% FBS) was dispensed into each well of the 24-well transwell plates and incubated at 37°C for 1 h. The transwell inserts were placed in the bottom of wells containing pre-warmed chemo attractant. EPC cells were trypsinized and resuspended in EGM-2 growth medium with 1% FBS. 1 × 10^4^ cells (600 μl from the cell suspension) were added to each well. The transwell plates were then incubated at 37°C. The number of migrated cells was counted after 16 h. The medium in the inserts was next removed, and the membranes were washed twice in PBS. Transwell membranes were then fixed in cold methanol for 1 min, air dried and washed three times before staining for nuclei using DAPI for 1 min. Transwell membranes were then washed at least three times with generous amounts of PBS to remove excess stain. The non-migrated cells on the top side of the membranes were gently wiped off using wet cotton swabs. The membranes were left to air dry and carefully peeled off from the inserts and placed on microscopic slides, with the migrated cells facing up down, then mounted in DEPEX mounting medium. Quantification was carried out by imaging 10 random X10 high-power fields per membrane using an Olympus inverted light/fluorescent microscope (Model No. IX81) and the number of migrated cells was counted using Image J software.

### Western blot analysis

Cells (2.5 × 10^5^ cells/well) were cultured in 6-well plates, and protein was extracted as previously described. Equal amounts of protein were then separated by electrophoresis using gradient SDS-PAGE gel, and transferred to nitrocellulose membranes (Amersham). After blocking with 5% non-fat milk in a buffer containing Tris–HCl (20 mM, pH 7.5), NaCl (100 mM) and Tween 20 (0.1%), respective membranes were incubated at 4°C overnight with primary rabbit monoclonal anti-NOX4 (1:1,000, Abcam, Australia) and mouse monoclonal anti-GAPDH (1:1,000, Merck Millipore) antibodies. Proteins were detected using an enhanced chemiluminescence detection kit (GE Healthcare, New South Wales, Australia) with horseradish peroxidase conjugated to appropriate secondary antibodies (Bio-Rad, New South Wales, Australia). The image was captured and processed using CanoScan 8800F/PhotoStudio 5.5 software.

### Data and statistics

Data are expressed as mean ± standard error of the mean (SEM). The mean data were analyzed with Student's *t*-test or One-way analysis of the variance (ANOVA) followed by *post-hoc* Tukey or Dunnett analysis. A value of *P* < 0.05 was regarded as statistically significant.

## Results

### Characterization and expansion of EPCs

**Figure 1:**
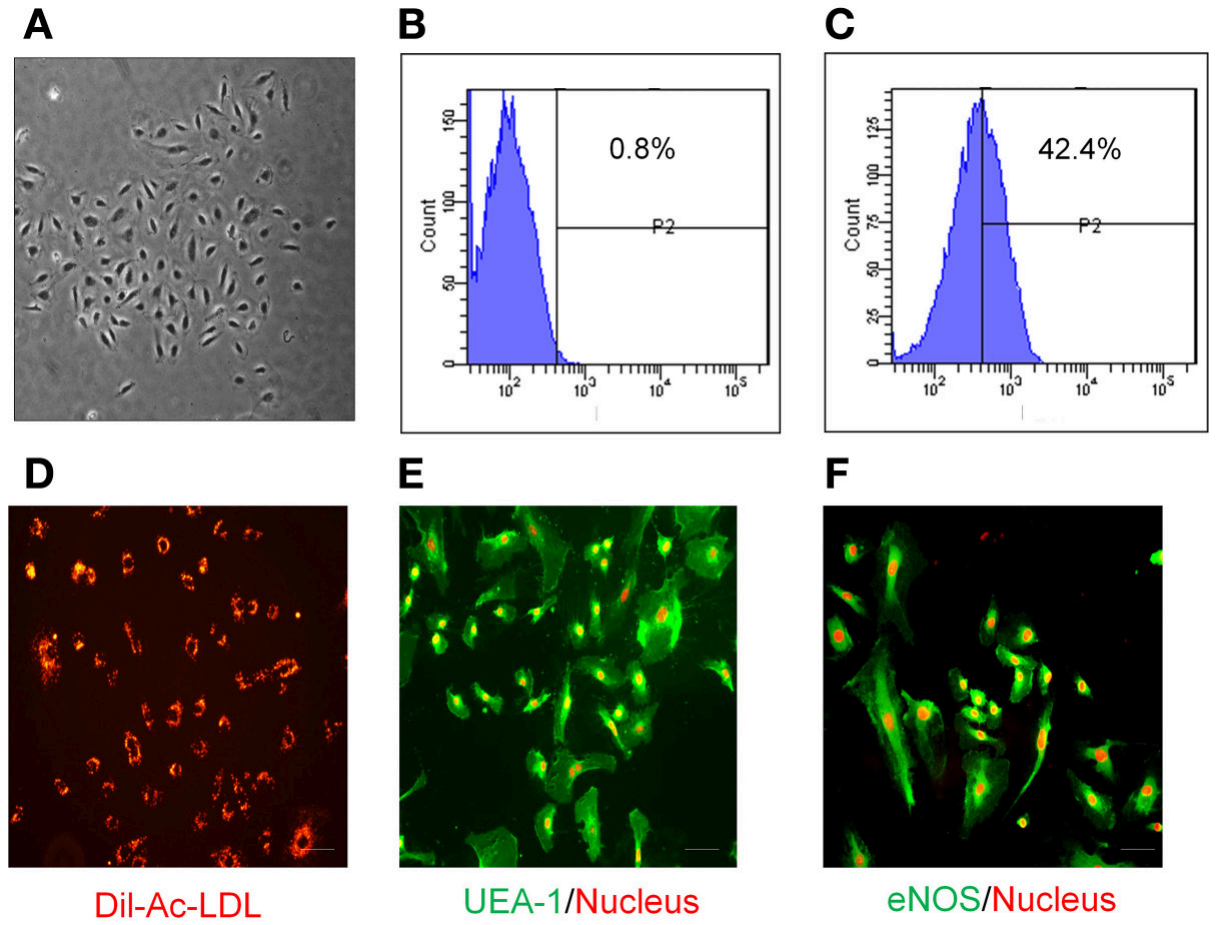
**Characterization of human EPCs. (A)** Bright field image of EPCs at low density (10^3^ cells/cm^2^) showing that they form colonies *in vitro* within 2 weeks. Compared with Isotype control **(B)**, FACS analysis of EPCs **(C)** at low density shows these cells express CD133. EPCs have the ability to uptake **(D)** Dil-Ac-LDL (10 μg/mL; Biomedical Technologies), and stain positive for both **(E)** UEA-1 (1:100; Vector Laboratories) and **(F)** eNOS (1:100; cell signaling). Propidium iodide (red; **E,F**) was used for nuclear staining. Images were taken by fluorescent microscopy (Scale bar = 10 μm).

EPCs have been successfully isolated and expanded in culture from different sources (Takahashi et al., 1999; Kalka et al., 2000; Yamaguchi et al., 2003; Ingram et al., 2004; Li et al., 2006). Here, EPCs were isolated from the luminal wall of human mammary artery/saphenous vein and expanded *in vitro* under low cell density culture conditions (10^3^ cells/cm^2^). EPCs formed colonies within 14 days (Figure 1A) and immunophenotyping showed high expression of hematopoietic marker CD133 at low density as compared to isotype controls (Figures 1B,C). Adherent EPCs stained positive for Dil-Ac-LDL (Figure 1D) and UEA-1 lectin (Figure 1E). Moreover, these cells stained positive for expression of the endothelial marker protein endothelial nitric oxide synthase (eNOS; Figure 1F).

**Figure 2:**
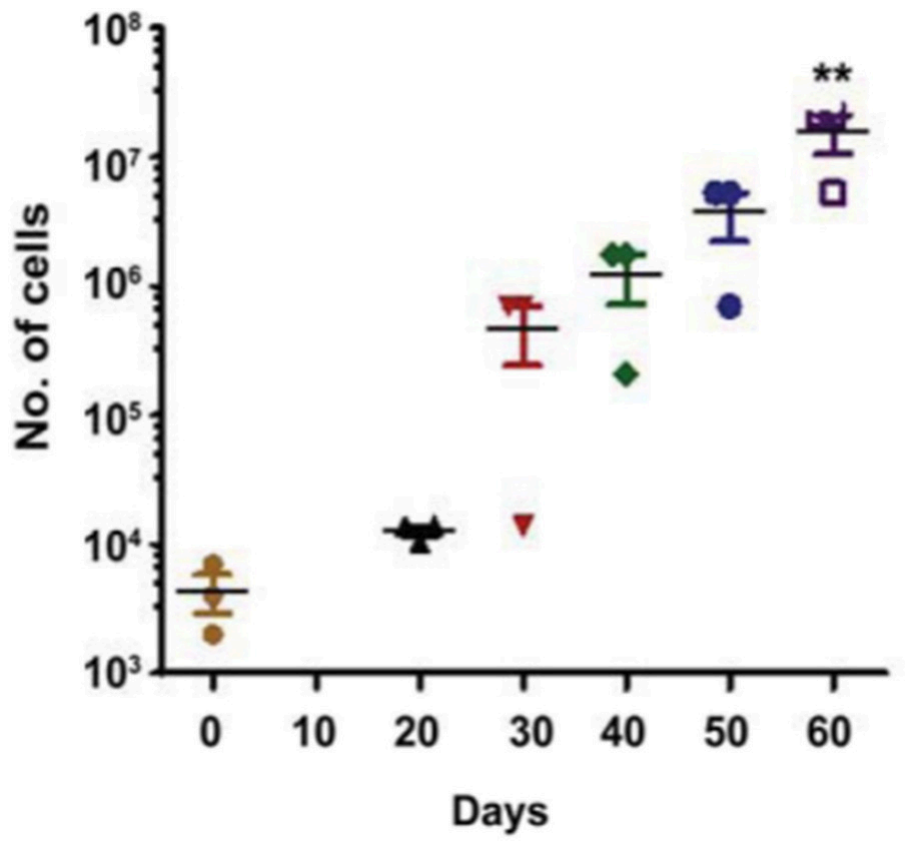
**Growth kinetics of the human isolated EPCs**. The figure shows a growth curve of isolated EPCs from three different patients (*N* = 3). Isolated EPCs show an initial lag phase of growth (20–30 days) before entering an exponential growth phase. Around 3,500-fold expansion was observed within 2 months of isolation with an average doubling time of 5.0 days. Values (means ± SEM; *n* = 3) are expressed as cell counts ^**^p < 0.05 from number of cells at day 0. Data were analyzed with one-way ANOVA followed by *post-hoc* Dunnett analysis.

Early and late EPCs can be distinguished by their proliferative property and life span in culture (Marui et al., 1993; Asahara et al., 1997; Hur et al., 2004). We examined proliferative potential of our expanded progenitor cells from three different patients. Cells were plated on day 0, and then serially passaged to maintain cells under proliferative phase for 60 days. Remarkably, colonies derived from EPCs started to appear from 20 days (Figure 2), suggesting that these cells are late EPCs. Every 10 days, the number of EPCs expanded until the induction reached around 3,500-fold within 60 days. This finding suggests that late EPCs can be expanded for a long time *in vitro*.

### Differentiation of EPCs to endothelial cells

**Figure 3:**
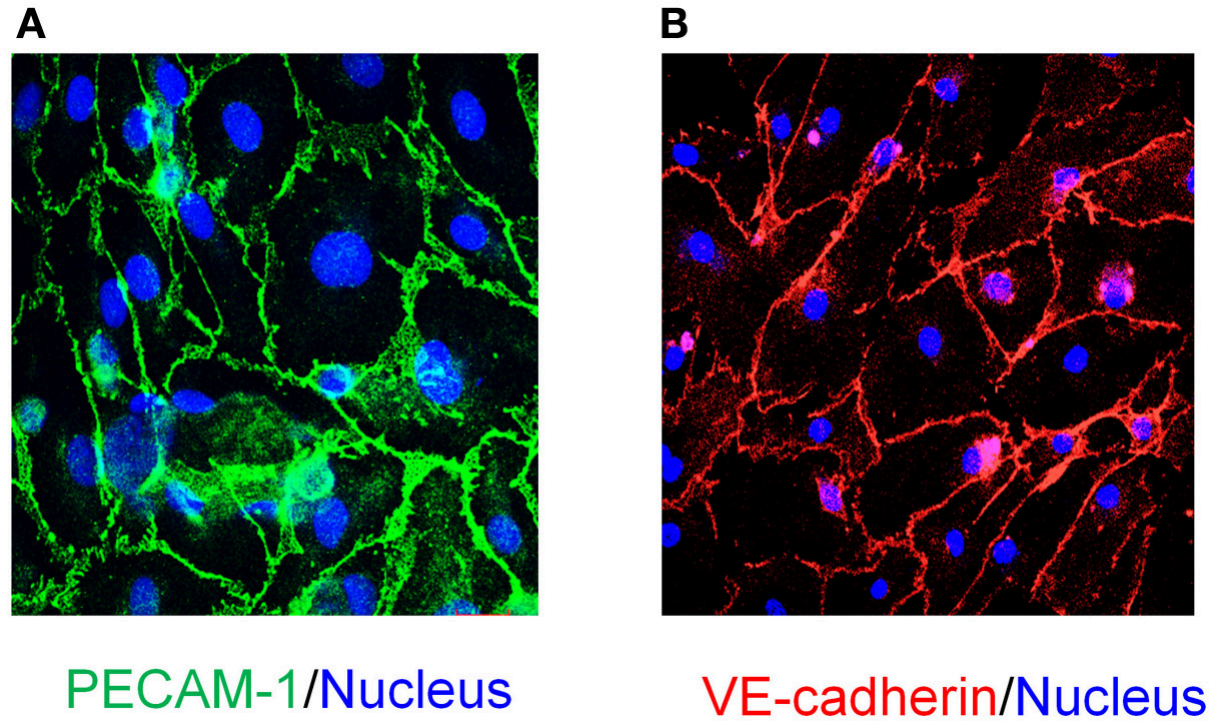
*****In vitro*** differentiation of EPCs to endothelial cells and characterization**. EPCs at high density (86 × 104 cells/cm^2^) differentiate into mature endothelial cells with cobblestone morphology as seen in **(A,B)**. Characterization of *in vitro* differentiated endothelial cells demonstrates that these cells are immunopositive for endothelial cell specific markers: **(A)** PECAM-1 (Scale bar 20μM) and **(B)** VE-cadherin (Scale bar 50μM). DAPI (blue; **A,B**) was used for nuclear staining (Scale Bar = 50 μm).

EPCs can be readily differentiated to endothelial cells. We examined the differentiation capacity of late EPCs which were plated at a high cell density (8 × 10^4^ cells/cm^2^) onto tissue culture treated chambers. Within 8 days late EPC had matured into endothelial cells which showed cobblestone morphology, as well as expressing endothelial markers PECAM-1 (Figure 3A) and VE-cadherin (Figure 3B).

### Nox4 is required for H_2_O_2_ generation in late EPCs

**Figure 4:**
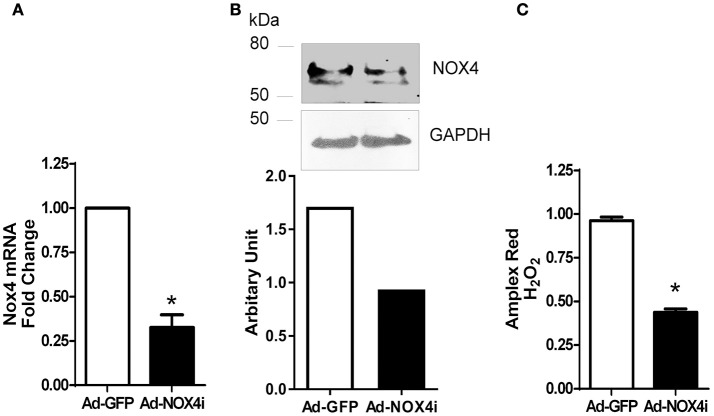
**Inhibition of NOX4 reduced NOX4 expression and H_**2**_O_**2**_ generation in late EPCs. (A)** Inhibition of NOX4 gene using NOX4 RNAi markedly reduced NOX4 mRNA expression in EPCs. **(B)** Lack of NOX4 decreased NOX4 protein level when compared to control in EPCs using a specific NOX4 antibody as shown in a representative Western Blot. **(C)** H_2_O_2_ generation was reduced after NOX4 inhibition using Amplex red. Values (means ± SEM; *n* = 3–5), ^*^P < 0.05 from Ad-GFP control following a Student's paired *t*-test.

To demonstrate the importance of NOX4 function in EPCs, adenovirus expressing RNA interference targeting human NOX4 (Ad-NOX4i) was used to suppress NOX4 gene expression. Inhibition of NOX4 using Ad-NOX4i (1,000 MOI) markedly reduced both NOX4 mRNA (Figure 4A) and protein (Figure 4B) expression compared with the control group (Ad-GFP). Moreover, Ad-NOX4i suppressed H_2_O_2_ production in late EPCs (Figure 4C), confirming that NOX4 promoted H_2_O_2_ generation in late EPCs.

### NOX4 is involved in EPC proliferation and migration

**Figure 5:**
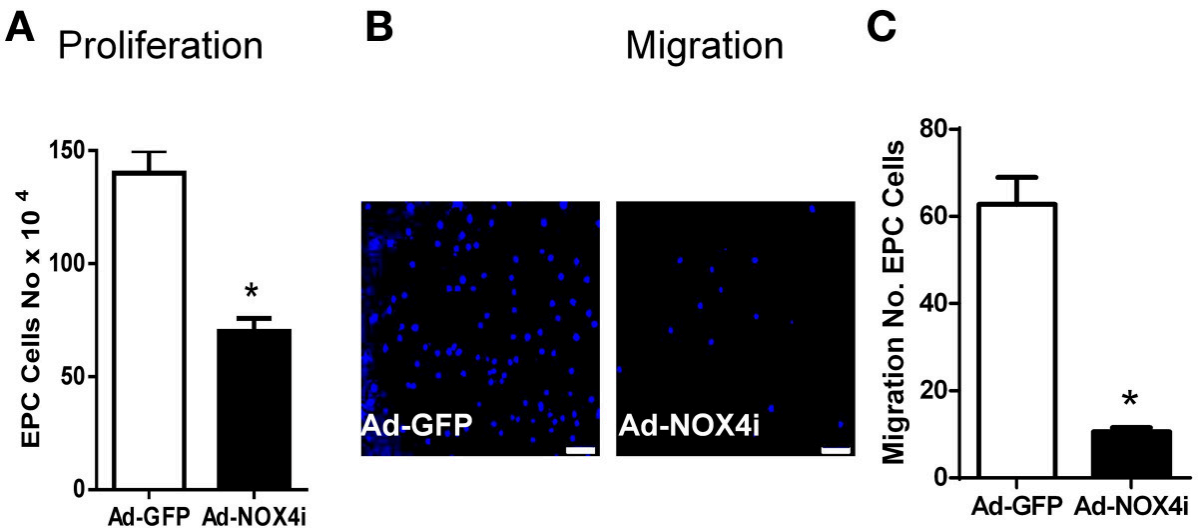
**Inhibition of NOX4 reduced proliferation and migration of late EPCs. (A)** EGM-2 growth medium was used to induce endothelial cell proliferation in Ad-GFP and ad-NOX4i. Cell proliferation was measured using trypan blue cell counting. Deficiency of NOX4 gene significantly reduced EPC proliferation compared with control (Ad-GFP). **(B)** Representative images showing a reduction in the number of migrated cells, stained with nuclear counter-stain DAPI after inhibition of NOX4 gene, compared with Ad-GFP control in the transwell migration assay (original magnification 40×) Scale bar = 10 μm. **(C)** Graph represents the quantitative data of Ad-GFP vs. Ad-NOX4i cell migration. Values (means ± SEM; *n* = 3–4) are expressed as cell counts, ^*^P < 0.05 from Ad-GFP control following a Student's paired *t*-test.

NOX4 has been also reported to mediate vascular cells proliferation and migration (Petry et al., 2006; Sturrock et al., 2006; Datla et al., 2007; Ismail et al., 2009; Peshavariya et al., 2009, 2014a,b). As shown in Figure 5A, EPC proliferation was clearly reduced after knocking down NOX4 using Ad-NOX4i when compared with control (Ad-GFP). We also examined the contribution of Nox4 on EPC migration using transwell migration assay. For this purpose, 1 × 10^4^ of Ad-GFP and Ad-NOX4i EPCs were seeded on the upper chamber of the transwell inserts and stimulated with EGM-2 growth medium + 1% FBS to pass to the lower chamber. The number of migratory cells was significantly decreased after inhibition of NOX4 gene when compared with the control (Ad-GFP) (Figures 5B,C). Thus NOX4 is required for proliferation and migration of EPCs.

### TNFα has a differential effect on expression of NOX2 and NOX4 isoforms in EPCs

**Figure 6:**
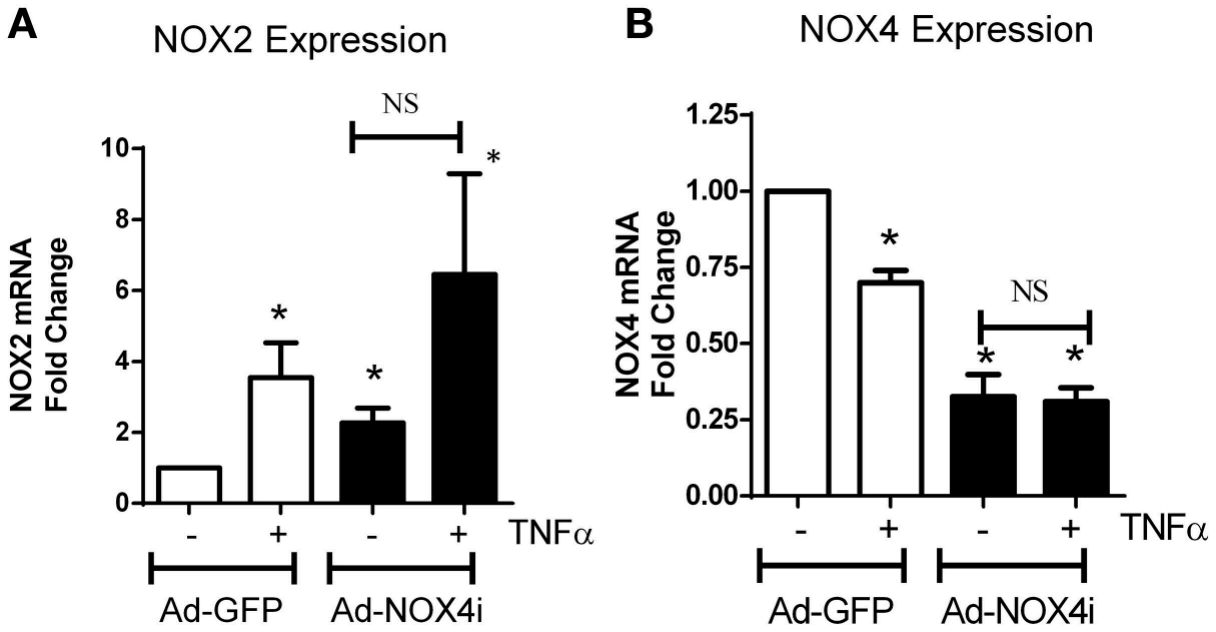
**TNFα has a differential effect on NOX isoform expression in EPCs. (A)** After TNFα treatment (20 ng/ml; 6 h), NOX2 mRNA levels increased in Ad-GFP cells and increased in NOX4 deficient cells. **(B)** Conversely, NOX4 mRNA expression was reduced in Ad-GFP infected cells and this was abolished in NOX4 deficient cells. Values (means ± SEM; *n* = 4) are expressed as a fold change compared with control (Ad-GFP), ^*^P < 0.05 from Ad-GFP control. Data were analyzed with one-way ANOVA followed by *post-hoc* Tukey analysis. NS: Not significant.

Pro-inflammatory cytokines such as TNFα, induce to oxidative stress via escalating ROS production (Kim et al., 2010) particularly from NADPH oxidase (Chandel et al., 2001). TNFα up-regulated the expression of NOX2 over 6 h, which was further induced in NOX4- deficient cells (Figure 6A). In contrast, NOX4 expression decreased after TNFα treatment. There was no significant difference in NOX4 expression before and after TNFα treatment in NOX4 deficient cells (Figure 6B), indicating that TNFα selectively modulates NOX isoforms in EPCs.

### NOX4 protects EPCs from cell death

**Figure 7:**
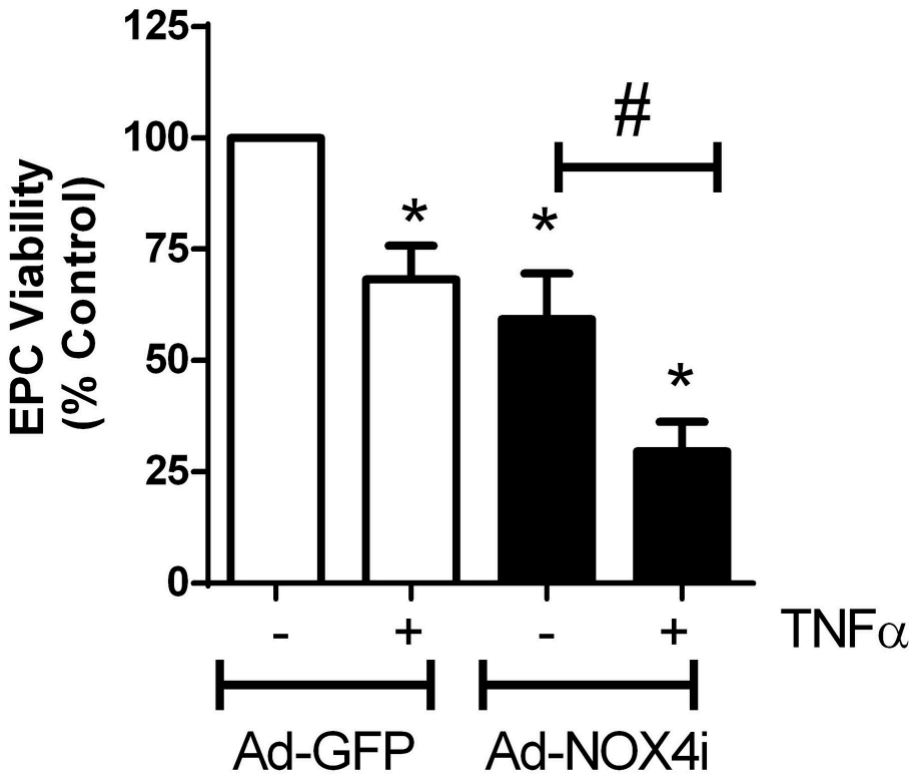
**NOX4 protects EPCs from TNFα-induced cell death**. Treating EPCs with TNFα (20 ng/ml; 48 h) induced cell death, which was further enhanced with NOX4 gene inhibited. Values (means ± SEM; *n* = 3–5) are represented as a percentage of control, ^*^P < 0.05 from Ad-GFP control, ^#^*P* < 0.05 from Ad-NOX4i. Data were analyzed with one-way ANOVA followed by *post-hoc* Tukey analysis.

Ad-GFP carrying EPCs treated with TNFα (20 ng/ml) for 48 h significantly increased cell death. Suppression of NOX4 gene using NOX4 RNAi in the absence of TNFα also increased cell death. Interestingly, the effect of TNFα on cell death was further enhanced in NOX4 deficient cells (Figure 7), indicating that NOX4 helps to protect EPCs.

## Discussion

We isolated highly proliferative CD133+ late EPCs from blood vessels and differentiated them into mature endothelial cells. We showed that NOX4 type NADPH oxidase is constitutively active and generates H_2_O_2_ in EPCs, whilst suppression of NOX4 reduced both their proliferation and migration. The pro-inflammatory cytokine TNFα reduced NOX4 expression and induced EPCs death. These finding suggest that endogenous NOX4 NADPH oxidase is important for EPC proliferation and migration and protects the cells under inflammatory conditions.

EPCs play a vital role in vascular homeostasis through re-endothelialization and neovascularization (Asahara et al., 1999a; Assmus et al., 2002; Iba et al., 2002; Griese et al., 2003; Urao et al., 2008; Schröder et al., 2009, 2011). Although, EPCs have been widely investigated for more than a decade, it is unclear how this cell population should be defined since no unique marker has been identified. During embryonic development, progenitors cells such as haemangioblasts give rise endothelial cells. These precursor cells are characterized by CD133 expression (Peichev et al., 2000), are highly proliferative and mediate an important role of ischemic tissues regeneration as well as repair of damaged blood vessels repair (Kocher et al., 2001). We and others have shown that CD133+ EPC populations can be isolated and expanded from various tissues such as blood vessels (Ranjan et al., 2009), heart (Beltrami et al., 2003), peripheral blood (Asahara et al., 1997), umbilical cord blood (Murohara et al., 2000) and bone marrow (Shi et al., 1998). Consistent with our previous study (Ranjan et al., 2009), blood vessel- derived EPCs retained the haematopoietic stemness marker CD133 in low density culture conditions and formed large cell colonies, take up Di-Ac-LDL, are positive for UEA-1 conjugated FITC binding and eNOS expression, and these features define EPC lineages. In addition to cell surface marker characteristics, EPCs are also characterized by certain biological properties such as proliferation and life span in culture conditions. It has been shown that haematopoietic early EPCs exhibit peak growth within 2 weeks and die by 4 weeks, whereas, late EPCs have a cobblestone-like morphology, they exhibit exponential growth at 4–8 weeks and can survive for up to 12 weeks (Marui et al., 1993; Asahara et al., 1997; Hur et al., 2004). Similarly, we observed that blood vessel- derived EPCs appeared cobblestone-like in morphology (Figure 1A) and proliferated exponentially after 3 weeks (Figure 2). Furthermore, EPCs spontaneously differentiated into endothelial cells under high density culture conditions and stained positive for CD31 and VE-cadherin (Figures 3A,B). These findings suggest that the human vascular wall retains CD133+ late EPCs that can be expanded *in vitro*.

NADPH oxidase-derived ROS signaling has been implicated in stem or progenitor cell function. More specifically ischemia mediated NOX2- derived ROS play a critical role in early EPC function such as mobilization, homing, and angiogenic capacity, thereby promoting revascularization of ischemic tissue (Urao et al., 2008) and re-endothelialization of injured blood vessels (Schröder et al., 2009). A later study also indicates that the NOX2 expression is higher than other NOX isoforms in human blood derived early EPCs and can be further stimulated in the presence of erythropoietin or under hypoxic conditions. In the present study, we demonstrated that NOX4 is an important source of H_2_O_2_ in CD133+ late EPCs. Moreover, inhibition of NOX4 reduces proliferation and migration of EPCs which are vital for angiogenesis (Figure 5). As with EPCs, we and others have shown that NOX4- deficient endothelial cells exhibit reduced *in vitro* proliferation and migration as well as *in vivo* angiogenesis (Petry et al., 2006; Datla et al., 2007; Peshavariya et al., 2009, 2014a; Craige et al., 2011). This functional similarity may be due to hierarchal preservation of NOX4 signaling in late EPCs as they mature.

The precise mechanisms by which NOX4 modulates functional activities of EPCs remain to be determined. However, previous studies have demonstrated that NOX4-derived H_2_O_2_ targets several proteins such as protein-tyrosine phosphatase 1 B (PTP1B) (Chen et al., 2008) and hypoxia-inducible factor 1-alpha (HIF1-α) (Zhang et al., 2010). Indeed, we and others have shown that NOX4 inhibits PTP1B and enhanced VEGF mediated cell signaling pathways in endothelial cells (Datla et al., 2007; Chen et al., 2008). A separate study has also revealed that NOX4 stabilizes HIF1-α and enhances VEGF expression (Meng et al., 2012). Elevation of growth factor expression and activity or both may enhance the function of EPCs in an autocrine or paracrine fashion and such a downstream target of NOX4 in EPCs needs to be explored in the future.

In contrast to NOX4 isoform-derived reductive stress, a sustained increase in oxidative stress due to aging and chronic pathological conditions such as diabetes and inflammation may impact on EPC numbers and functions (Ebrahimian et al., 2006; Hamed et al., 2009). Indeed, many lines of evidence indicate that NOX2-derived oxidative stress reduces circulating EPCs in diabetic patients as well as hampering the neovascularization efficiency of EPCs in an experimental model of hind limb ischaemia in aged mice (Turgeon et al., 2012). Moreover, separate studies show that the pro-inflammatory cytokine TNFα also activates NOX2 and reduces the viability of endothelial cells (Frey et al., 2002; Li et al., 2005; Schroder et al., 2012) and EPCs (Du et al., 2014). Consistent with these studies, we found chronic exposure to TNFα induces NOX2 expression and decreases survival of EPCs. Interestingly, TNFα decreased the expression of NOX4 and further reduced EPC viability in NOX4 deficient cells suggesting that NOX4 provides protective signaling which is important for EPC survival. Although precisely how TNFα reciprocates NOX isoform expression in EPCs remains to be identified. In addition, Nox isoforms expression also altered as diseases progress which may lead to dysfunctionality of EPC. Indeed Zhang et al. shown that EPC obtained from coronary artery diseases patients have reduced migration and angiogenic activity compared to healthy subjects due to activation of p47Phox subunit (required for Nox2 and Nox1 isoforms; Zhang et al., 2016). In the present study, we have isolated EPC from blood vessels derived from cardiac surgery patients and did not compared with healthy subjects. It is a limitation of present work and such a comparison study required to identify the precise role of Nox4 in EPC.

In conclusion, we found that NOX4 type NADPH oxidase is constitutively active and generates H_2_O_2_ in CD133+ late EPCs. In addition, NOX4 is important for proliferation, migration and survival of EPCs which are vital for neovascularization. Recently we have shown that a prostacyclin analog can increase NOX4 expression in endothelial cells and EPCs and promotes angiogenesis *in vitro* and *in vivo* (Peshavariya et al., 2014b). Collectively, these findings suggest that targeting NOX4 in EPCs may be a therapeutically feasible approach to enhance the repair of damaged blood vessels and neovascularization.

## Author contributions

NH, AR, and HP, Conceived the study and wrote the manuscript. AH and GD: Provided reagents and intellectual inputs. All authors read and approved the final manuscript.

### Conflict of interest statement

The authors declare that the research was conducted in the absence of any commercial or financial relationships that could be construed as a potential conflict of interest.
