# Supplementary material for: Role of NADPH Oxidase-4 in Human Endothelial Progenitor Cells
Source: Front Physiol. 2017 Mar 23;8:150. doi: 10.3389/fphys.2017.00150 (PMC5362645; doi:10.3389/fphys.2017.00150)
Supplement: Supplementary file 1 [file DataSheet1.DOCX]

| **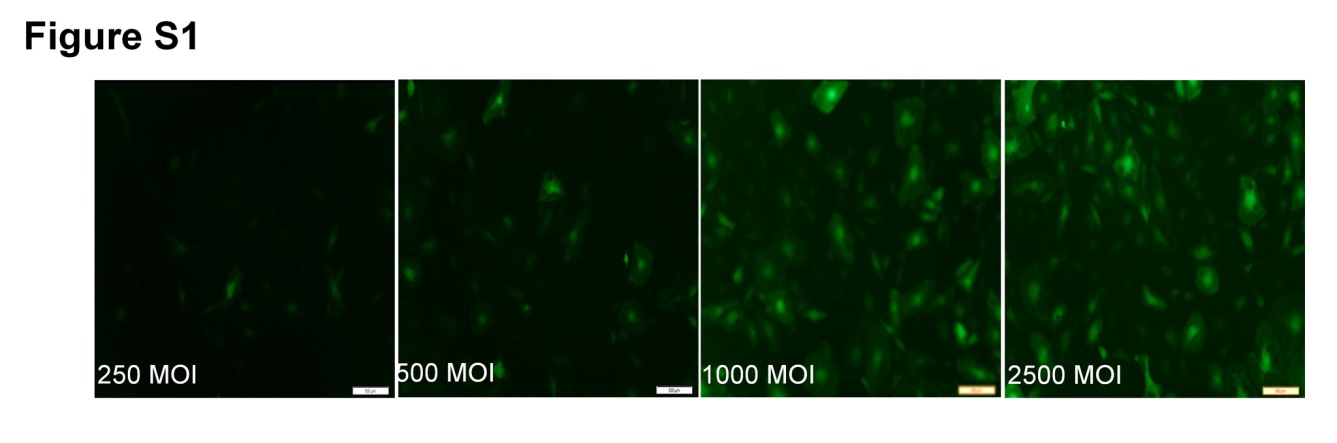** |
| --- |

**Supplementary Figure S1:** M**ultiplicity of infection (MOI) for Ad-GFP infected EPCs.**

EPCs (5x10^4^) were cultured in a 10 cm^2^ plate one day before the infection with different titres of Ad-GFP (MOI 250-2500) for 24h to determine the efficiency cell infection. Positive infected cells expressed green fluorescence under fluorescence microscopy (Zeiss AxioImager.2 microscope). Scale Bar = 50 µm.
